# Supplementary material for: Prognostic risk models for incident hypertension: A PRISMA systematic review and meta-analysis
Source: PLoS One. 2024 Mar 11;19(3):e0294148. doi: 10.1371/journal.pone.0294148 (PMC10927109; doi:10.1371/journal.pone.0294148)
Supplement: S1 Appendix — (DOCX) [file pone.0294148.s002.docx]

# Search phrases

For PubMed, the following key word combination was used:
*(hypertension[Title/Abstract] OR ”high blood pressure”[Title/Abstract]) AND (”prediction model”[Title/Abstract] OR ”risk model”[Title/Abstract] OR ”risk equation”[Title/Abstract] OR ”risk score”[Title/Abstract]) AND (english[Filter])*.

For Web of Science, the following key word combination was used:
*TS**=((hypertension* *OR ”high blood pressure”) AND (risk OR predic*) AND (model OR equation OR score)) AND TI=((hypertension OR ”high blood pressure”) AND (predic* OR risk)).*

The reference lists of each included article and works citing the included articles were also searched for any eligible articles using Google Scholar.

# Sensitivity analysis of mis-specifying sample-error covariance in meta-analyses

Model results originating from the same study and datasets may be correlated. However, we did not find any covariances or correlations for sample-errors in the included studies, and subsequently assumed sample-error correlations were zero for our meta-analysis.

To assess the possible impact of this assumption, we redid the meta-analyses of ML and traditional models with simulated sample-error covariances between results from the same studies. In practice, for each study, we sampled one common sample-error correlation from a uniform distribution on the interval 0 to 1 and imputed sample-error covariances for the results using the sampled correlation. We then applied the meta-analyses again and recorded the intra-class correlation, I^2^, heterogeneity estimate (Tau) and the pooled effect. We repeated the sensitivity analysis one thousand times to obtain reliable parameter distributions.

Parameter estimates found in our sensitivity analysis proved to be close to those obtained by ignoring sample-error correlation, shown in Table 1, implying negligible impact for our assumption. A possible explanation is the large difference in the scale of heterogeneity compared to the sample-errors covariances that were ignored: Tau was estimated in our meta-analyses to be 7 and 5.5 times larger than the highest possible sample-error covariance for traditional regression and machine learning models, respectively. In truth, the possible sample-error covariance is far lower for most data, and it is unlikely that they are perfectly correlated, meaning the covariance would be even lower. Simply put, the estimated heterogeneity between studies is far higher than any possible sample-error covariance omitted in our analysis.

**Table 1. Meta-analysis parameters after random simulation of sample-error covariances.**

| Traditional regression-based models | Assuming no sample-error covariance | With randomly sampled sample-error covariances, 95% confidence interval |
| --- | --- | --- |
| Intra-class correlation | 90.38% | [88.60 – 90.35] % |
| I^2^ | 99.05% | [99.0512 – 99.0514] % |
| Tau | 0.0846 | [0.0846 – 0.0862] |
| Pooled effect | 0.7792 | [0.7792 – 0.7792] |
|  |  |  |
| Machine learning models | Assuming no sample-error covariance | With randomly sampled sample-error covariances, 95% confidence interval |
| Intra-class correlation | 60.41% | [60.09 – 60.40] % |
| I^2^ | 99.85% | [99.846 – 99.847] % |
| Tau | 0.3403 | [0.3403 – 0.3408] |
| Pooled effect | 0.81702 | [0.81703 – 0.81706] |
