# Supplementary figures and images for: Prognostic risk models for incident hypertension: A PRISMA systematic review and meta-analysis
Source: PLoS One. 2024 Mar 11;19(3):e0294148. doi: 10.1371/journal.pone.0294148 (PMC10927109; doi:10.1371/journal.pone.0294148)

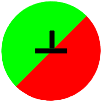

Supplement: S1 File — (ZIP) [file pone.0294148.s003.zip › S1_File/partial_bias_circle.png]
